# Preliminary study of the impact of elevated circulating plasma levels of catecholamines on opioid requirements for acute surgical pain

**DOI:** 10.1017/cts.2020.573

**Published:** 2021-01-05

**Authors:** Armando Uribe-Rivera, Linda Rasubala, Ana C. Machado-Perez, Yan-Fang Ren, Hans Malmström, Adam Carinci

**Affiliations:** 1 Howitt Urgent Dental Care Department, University of Rochester, School of Medicine and Dentistry, Rochester, NY, USA; 2 General Dentistry Department, University of Rochester, School of Medicine and Dentistry, Rochester, NY, USA; 3 Department of Anesthesiology & Perioperative Medicine, University of Rochester Medical Center, Rochester, NY, USA

**Keywords:** Opioids, acute pain, catecholamines, morphine milligram equivalents, postoperative pain

## Abstract

**Introduction:**

The objective of this study is to determine whether elevated circulating plasma catecholamine levels significantly impact opioid requirements during the first 24 hours postoperative period in individuals with acute surgical pain.

**Methods:**

We retrospectively reviewed 15 electronic medical records (EMRs) from adults 18 years and older, with confirmed elevated plasma catecholamine levels (experimental) and 15 electronic health records (EHRs) from matched-controls for age, gender, race and type of surgery, with a follow up of 24 hours postoperatively.

**Results:**

The total morphine milligram equivalents (MMEs) requirements from the experimental group were not statistically different when compared with controls [44.1 (13 to 163) mg versus 47.5 (13 to 151) mg respectively; p 0.4965]. However, the intraoperative MMEs showed a significant difference, among the two groups; [(experimental) 32.5 (13. to 130) mg, (control) 15 (6.5 to 130) mg; p 0.0734]. The intraoperative dosage of midazolam showed a highly significant positive correlation to the total MMEs (p 0.0005). The subjects with both elevated plasma catecholamines and hypertension used significantly higher intraoperative MMEs compared to controls [34.1 (13 to 130) mg versus 15 (6.5 to 130) mg, respectively; p 0.0292)]. Those 51 years and younger, with elevated circulating levels of catecholamines, required significantly higher levels of both the postoperative MMEs [29.1 (0 to 45) mg versus 12 (0 to 71.5) mg; (p 0.0553)] and total MMEs [544.05 (13 to 81) mg versus 29.42 (13 to 92.5) mg; (p 0.00018), when compared to controls with history of nicotine and alcohol use.

**Conclusion:**

This preliminary study evaluated a biologic factor, which have promising clinical usefulness for predicting analgesic requirements that can drive clinical decisions on acute surgical pain.

## Introduction

Opioids are commonly used to control acute postoperative pain. However, opioids are associated with some drawbacks, including adverse side effects related to higher opioid requirements and subsequent opioid use disorders [1]. It is estimated that ∼90 million individuals in USA have received a prescription for an opioid. Of these, ∼3 million users develop an opioid use disorder [2,3]. These opioid-related issues correspond to an economic burden of ∼$78.5 billion USD, linked to costs associated with increased emergency department visits, health care problems, substance abuse treatments, and increased burdens on the judicial system [3,4].

Currently, postoperative opioid research has identified several factors among individuals, excluding pharmacogenomics, which may impact their opioid therapy response [5]. On one hand, non-genetic factors, including depression, anxiety, and pre-existing history of substance abuse, are associated with altered pain perception and higher requirements of analgesic medication for pain control [6–8]. On the other hand, biologic and genetic factors, including the endogenous opioids (enkephalins, *B*-endorphins, dynorphins) along with catecholamines (epinephrine, norepinephrine, dopamine), are praised to be major components in pain perception and analgesic requirements [9,10]. For instance, the assumed role of the abnormal pool of catecholamines in adrenergic neurons in the population with genetic variants of major catecholamine enzymatic pathways is believed to predispose these individuals more often to request an analgesic drug for pain relief, receiving higher milligrams of pain medication for acute surgical pain control [11–13].

Although, the above scientific evidence has expanded our understanding of the pain response and its relationship to key molecule signaling pathway, including the catecholamines, further translational research is needed to demonstrate the clinical relevance of these adrenergic components and their impact in the management of acute surgical pain. In addition, further research will elucidate how practical and clinically useful these biologic factors are in 1) identifying the population most vulnerable for requesting excessive dosages of opioid drugs and 2) driving clinical decisions to select alternative analgesic therapies, to prevent future opioid use disorders.

Therefore, the purpose of this preliminary study is to evaluate whether opioid requirements for acute surgical pain, require significantly higher MMEs during the first 24 hours postoperative period, compared to controls. Our two secondary hypotheses include 1) “Intraoperative pain therapies that includes midazolam, propofol, and 2% lidocaine, significantly, impact the requirements of MME during the first 24 hours postoperative period in adults with elevated levels of circulating plasma catecholamines and acute surgical pain, compared to controls” and 2) “adults with elevated levels of catecholamines and acute surgical pain, require similar MMEs during the first 24 hours for pain management, compared to those with normal levels of catecholamines, who have pre-existing psychiatric comorbidities.”

## Methods

### Study Design

Under the auspices of the Institutional Review Board at the University of Rochester (IRB#00004557), a retrospective cohort matched-control for age, gender and type of surgery study was designed. Patients in the experimental group had diagnosis of pheochromocytoma (study model for elevated circulating catecholamine levels in the blood) [14], with acute surgical pain treated with opioid therapies in a hospital setting. Subject’s electronic medical records (EMRs) were followed for 24 hours after surgical intervention. The primary outcome variable was total MMEs during the first 24 hours postoperative period. Secondary outcome variables were intraoperative pain management in milligrams and other pre-existing medical problems, including diabetes, hypertension, depression, anxiety, fibromyalgia, and nicotine and alcohol use.

### Subject Selection

In collaboration with the Computational Informatics Department, at the Clinical and Translational Institute of the University of Rochester, we used TriNetX, an electronic database warehouse, which provided access to EMRs from patients at The University of Rochester Medical Center. We analyzed the EMRs of males and females, age 18 and older, who had an international code disease 9/10 [ICD9/10 codes] including medulloadrenal hyperfunction (ICD9-255.6), adrenomedullary hyperfunction (ICD10-E27.5), acute postoperative pain (ICD9-338.18), and other acute post-procedural pain (ICD10-G89.18) for identifying subjects in the experimental group. As a comparison group (controls), we selected patients who had ICD9/10 codes, including both ICD9-338.18 and ICD10-G89.18 only. We excluded all EMRs in the query that included chronic pain syndrome (ICD10-G89.4) and liver disease unspecified (ICD10-K76.9). Other demographic data included age at time of surgery, gender, height, weight, and body mass index (BMI). Other data included type of surgery and milligrams of opioid therapies converted into MMEs.

Those with elevated circulating levels of catecholamines were confirmed by laboratory results, for elevated metanephrine and normetanephrine levels in the blood and compared with controls. Of note, to confirm that elevated circulating catecholamine levels were not present in the control group, the medical informatics specialist identified those subjects who included the ICD9 and ICD10 codes aforementioned above only without the ones used for the experimental group. This is to purify our control group sample. We anticipated controlling for bias using a control-matched strategy for age, sex, race, and type of surgery.

### Follow Up

We reviewed EMRs of subjects with acute surgical pain who were treated with opioid therapies during the first 24 hours of hospitalization.

### Outcome Variables

The primary outcome variable was total amount of opioid therapy milligrams, converted to MMEs. Opioid medication therapy data was available in the EMRs, under the nursing notes in the floor and discharge summary of each study subject.

Secondary outcome variables included milligrams administered of intraoperative midazolam, propofol, and 2% lidocaine (as local anesthetic). Other variables included pre-existing medical conditions including hypertension, diabetes mellitus type 2, depression, anxiety, fibromyalgia, and nicotine and alcohol use.

### Statistical Analysis

The analysis of the primary outcome variable, total MMEs in the first 24 hours after surgery, involved a Wilcoxon rank-sum test. In addition, intraoperative and postoperative MMEs involved the Wilcoxon rank-sum test. For the analysis of the secondary outcome variables including; milligrams of intraoperative midazolam, propofol and 2% lidocaine and pre-existing medical problems, both the linear regression analysis and the Mann–Whitney–Wilkoxon U test were used. All statistical analyses used a two-tail test at a significance level of ≤0.1. Of note, as a preliminary study designed was selected, both the sample size and the effect difference were not determined.

## Results

**Fig. 1.**
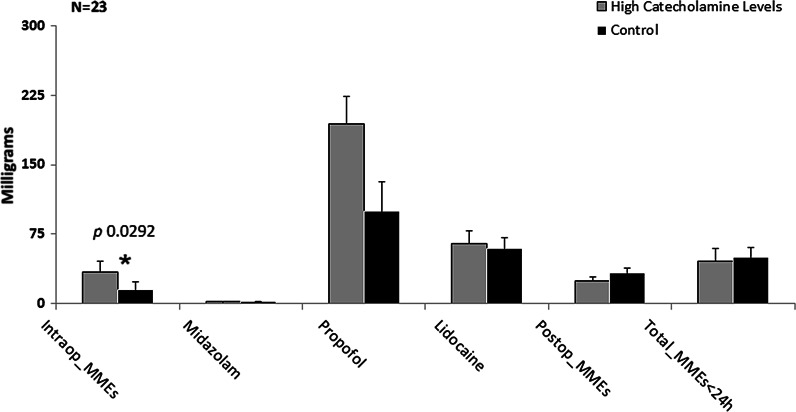
TriNetX search strategy. Flowchart representing the search of electronic medical records in TriNetX. MME, morphine milligram equivalents.

Of the 19,341 EMRs, identified from TriNetX, we removed 10,729 duplicates. From the first screening of 8612 EMRs, we excluded 1569 EMRs. From second screening of 7043 EMRs, 83 EMRs met inclusion criteria for experimental group. In addition, 6960 EMRs met inclusion criteria in the control group. The final screening of 7013 EMRs excluded 68 EMRs in the experimental group and 6945 EMRs in the control group. Thirty subject’s EMRs were included in the analysis (Fig. 1).

### Demographics and Pre-Existing Conditions

Among the 30 EMRs, 19 were females and 11 males. There were no significant differences in demographics between the experimental group and controls. The Median (range) age in the experimental group was 52 (23 to 82) years compared to 59 (23 to 82) years of age in the controls (p 0.8807). Median height was 164 (154 to 187) cm in the experimental group compared to 167 (154 to 193) cm in the controls (p 1.0000). Median weight in the experimental group was 83.9 (43.1 to 158) kg compared to 78.3 (52 to 117.4) kg in the controls (p 0.4413). Median BMI in the experimental group was 27.7 (16.9 to 58.7) kg/m^2^ compared to 25.2 (15.8 to 39.2) kg/m^2^ (p 0.2891) in the controls. The proportion of pre-existing medical conditions in both the experimental group and the controls are listed in Table 1. This included 26.7% versus 33.3% with diabetes, 80% versus 73.3% with hypertension, 26.7% versus 26.7% with asthma, 60% versus 66.7% with depression, 33.3% versus 53.3% with history of nicotine and alcohol use, 40% versus 20% with anxiety, and 13.3% were found with fibromyalgia in the control group only.

**Table 1.** Demographics, comorbidities, and morphine milligram equivalents consumption. Table demonstrating the data on sample size (*N*), median, ranges, and proportions. Wilcoxon rank test sum test significance at *P* ≤ 0.1

Demographics	Elevated Plasma Catecholamines			Control			*P* value
	*N*	Median	Range	*N*	Median	Range	
Age	15	52	23 to 82	15	59	23 to 82	0.8807
Height (cm)	15	164	154 to 187	15	167	154 to 193	1.0000
Weight (kg)	15	83.9	43.1 to 158	15	78.3	52 to 117.4	0.4413
BMI (kg/m^2^)	15	27.7	16.9 to 58.7	15	25.2	15.8 to 39.2	0.2891

Comorbidities	*N*	Proportion		*N*	Proportion		
Diabetes (%)	15	26.7		15	33.3		
Hypertension (%)	15	80		15	73.3		
Asthma (%)	15	26.7		15	26.7		
Depression (%)	15	60		15	66.7		
Hystory of substance abuse (%)	15	33.3		15	53.3		
Anxiety (%)	15	40		15	20		
Fibromyalgia (%)	15	0		15	13.3		

Morphine Milligram Equivalent (MME)	*N*	Median	Range	*N*	Median	Range	*P* value
Introperative_MME	15	32.5	13 to 130	15	15	6.5 to 130	0.0734*
Postoperative_MME	15	25	0 to 45	15	24.1	0 to 71.5	0.4965
Total_MME ≤24 hours	15	44.1	13 to 163	15	47.5	13 to 151	0.4965

Other Intraoperative Medication	*N*	Median	Range	*N*	Median	Range	*P* value
Midazolam (mg)	15	2	0 to 2	15	2	0 to 9	**0.1770**
Propofol (mg)	15	199.2	12 to 300	15	120	0 to 450	0.9521
Lidocaine (mg)	15	80	0 to 100	15	80	0 to 100	0.5485

### Intraoperative Pain Management

The median and range of midazolam was 2 (0 to 2) mg in the experimental group compared to 2 (0 to 9) mg in the control group (p 0.1770). Median propofol was 199.2 (12 to 300) mg in the experimental group compared to 120 (0 to 450) mg in the control group (p 0.9521). The median of 2% lidocaine received in the operating room was 80 (0 to 100) mg for both the experimental group and control group (p 0.5485). (Table 1)

### Primary Outcome: Total MMEs

The results of the analysis for the comparison of MMEs, between the experimental and control groups, demonstrated that there was a significant difference in the amount of intra-operative MMEs between the groups [32.5 (13. to 130) mg versus 15 (6.5 to 130) mg, respectively; p 0.0734]. However, the amount of postoperative MMEs and total MMEs was not significantly different between the two groups [25 (0 to 45) mgs versus 24.1 (0 to 71.5) mg (p 0.4965) and 44.1 (13 to 163) mg versus 47.5 (13 to 151) mg, respectively (p 0.4965)]. (Table 1)

### Secondary Outcome 1: Intraoperative Pain Management and Total MMEs

The regression analysis results on the impact of intraoperative pain management on total MMEs, administered during the first 24 hours postoperative period, showed that propofol and 2% lidocaine had a negative correlation on total MMEs. However, this correlation was not significant (p 0.77 and p 0.69). On the other hand, midazolam showed a highly significant positive correlation to total morphine milligram equivalents (p 0.0005). (Table 2)

**Table 2.** Intraoperative pain therapy and total morphine milligram equivalents. Table representing regression analysis results. Significant level at *P* ≤ 0.1

Parameter	Estimate	Standard error	*P* value
Group C versus E	−15.818	12.365	0.20
Midazolam	12.389	3.576	0.0005
Propofol	−0.020	0.067	0.77
2% Lidocaine	−0.064	0.160	0.69

C, Control; E, Experimental groups.

### Secondary Outcome: Pre-Existing Medical Conditions and Total MMEs

Results from these analyzes showed that subjects with elevated circulating catecholamines and hypertension (*N* = 12), were administered with significantly higher intraoperative MMEs, compared to controls (*N* = 11) [34.1 (13 to 130) mg versus 15 (6.5 to 130) mg, respectively; p 0.0292) and significantly higher dosages of propofol [194.2 (12 to 300) mg versus 100 (0 to 450) mg, respectively; p 0.0687) (Fig. 1).

**Fig. 2.**
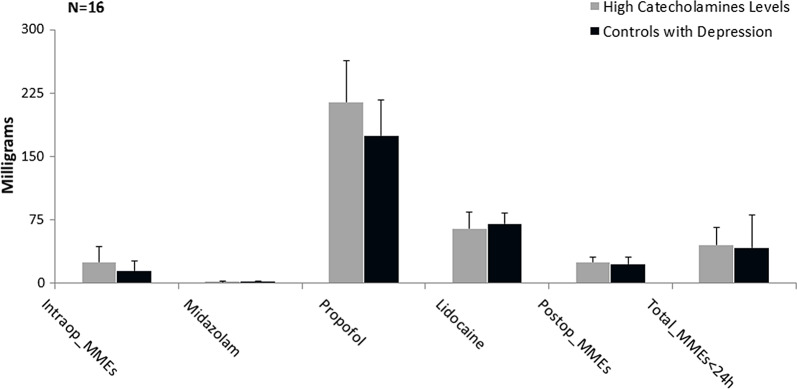
Comparison between experimentals with hypertension and controls for surgical pain therapy administered in milligrams. Graph represents the median and standard error of the milligrams administered for surgical pain medications. Mann–Whitney U test significant level at *P* ≤ 0.1. MME, morphine milligram equivalents.

**Fig. 3.**
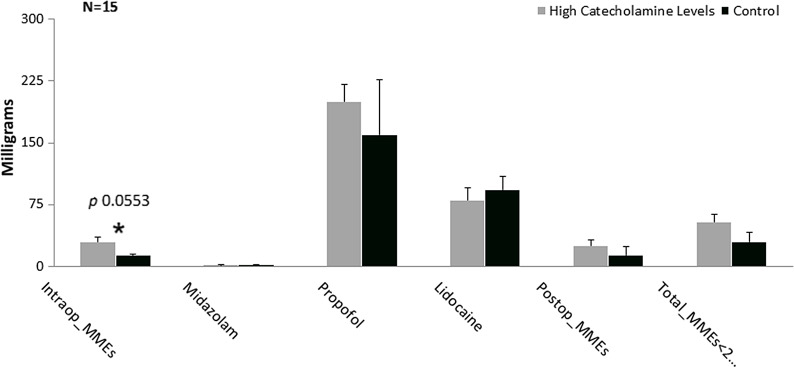
Comparison between those with elevated plasma catecholamines (experimentals) and controls with psychosocial abnormalities. Graphical representation of median and standard error of milligrams administered for surgical pain medications. Mann–Whitney U test significant level at *P* ≤ 0.1. MME, morphine milligram equivalents.

However, to test our secondary hypothesis that those with elevated circulating levels of catecholamines in the blood require similar MMEs when compared to other known conditions, which altered perception of pain and analgesic requirements. We compared those with psychosocial problems, including depression and history of nicotine and alcohol use in the control group, to subjects with elevated circulating catecholamines in the blood and no other psychosocial comorbidities in the experimental group. There was not a statistically significant difference between the two groups (total MMEs [44.1 (13 to 163) mg versus 47.5 (13 to 151.5) p 0.1936]). (Fig. 2) It is worth mentioning that those younger than 51 years of age, with elevated circulating levels of catecholamines, required significantly higher dosage of both the postoperative MMEs and the total MMEs when compared to controls with depression and history of nicotine and alcohol use [Postop_MMEs 29.1 (0 to 45) mg versus 13 (0 to 71.5) mg, respectively, (p 0.0553) and total_MMEs 54.05 (13 to 81.1) mg versus 29.42 (13 to 92.5) mg, respectively, (p 0.00018)] (Fig. 3).

## Discussion

The purpose of this preliminary study was to determine whether subjects with elevated circulating plasma catecholamines require higher levels of total MMEs during the first 24 hours postoperative period compared to controls. The findings in this study suggested that subjects with elevated circulating plasma catecholamines required higher intraoperative MMEs compared to controls. However, we could not find a significant difference between the two groups in the total MMEs, administered during the first 24 hours postoperative period. Moreover, most of our study population who did require higher intraoperative MMEs had pre-existing hypertension (>70%). It has been reported that ˜95% of the population with pheochromocytoma (study model in our experimental group) endures some type of hypertension secondary to the elevated levels of catecholamines (PMCID: PMC3094542). Similar to the outcomes in our study, previous research has highlighted the higher intraoperative opioid requirements in those with pre-existing hypertension. For instance, in a study from the Netherlands, it was reported that higher doses of intraoperative fentanyl (130–140 µg/kg) were administered to reduce hypertension on patients who underwent cardiac surgery [15]. Other reports by Stanley et al. demonstrated that those with intraoperative hypertension, secondary to the combination of smoking with other habits, including alcohol and caffeine use, required three times more intraoperative fentanyl to control the hypertension when compared to controls [16]. Correlating these results with our outcomes, we presumed that the significantly different intraoperative MME requirements among the subjects in the experimental group represented those who received intraoperative hypertension management with fentanyl as a protective cardiovascular strategy.

There is a safety concern about daily opioid requirements and acute surgical pain management in those most vulnerable for receiving higher MMEs for pain control. This is due to the impact of the elevated therapeutic drug requirements and its potential development of opioid use disorders [17]. Therefore, one of the secondary goals of the study was to determine the impact of intraoperative pain management on total MMEs administered during the first 24 hours postoperative period to control surgical pain. The findings in this study demonstrated that midazolam but neither propofol nor 2% lidocaine were highly correlated to higher total MMEs during the first 24 hours postoperative period. The dose range for the intraoperative midazolam administered was between 0 and 9 mg. Previous research has been shown that benzodiazepines potentially influence the MMEs of opioid therapies in the pain management population [18]. For instance, Suraya et al. reported that patients who received benzodiazepine therapies, combined with opioid drugs, received mean daily MMEs of 31.67 mg versus MMEs of <30 mg, associated with other combinations, including opioid +opioid and opioid +anticonvulsant therapies, to name a few [19,20]. Though, the findings in our study demonstrated similar results to Suraya’s study, there are some differences between Suraya’s study and ours. First, our study population had acute surgical pain only, while only 6.16% of the population from Suraya’s study had surgical or post-traumatic pain. Additionally, all subjects in our study received a combined intraoperative midazolam and fentanyl therapy, while only 12.5% and 7.1% of the subjects in Suraya’s study received fentanyl and midazolam, respectively. However, not reported were the number of patients who received both drugs for surgical pain management, making it difficult to determine the impact of this therapy on administered MMEs.

Other factors have been identified for predicting the patient’s responses to surgical pain therapies [19] Of these factors, a history of substance abuse and depression is acknowledged to alter pain perception and abnormal opioid drug consumption [21,22]. Cryar showed that the opioid management in patients with surgical pain secondary to knee and hip arthroplasty procedures and concurrent smoking status required higher MMEs than controls in the next 3 months after surgery. However, in Cryar’s study, the MMEs ranged between 250 and 5540 mg compared to 0 to 3600 mg in the control group [23]. In addition, other reports investigating the correlation between smoking and analgesic use during the first 12 hours postoperative period suggested that females who underwent pelvic surgery and reported smoking consumed ˜20% more opioid drugs for controlling postoperative pain (13 mg/12 hours versus 10.5 mg/12 hours reported by controls) [24]. In contrast, our findings suggest that there is not a statistical difference in total MMEs administered during the first 24 hours postoperative period between those with elevated circulating levels of catecholamines with no psychosocial issues compared to controls with psychosocial issues, including depression and history of nicotine and alcohol use. This analytic comparison demonstrated that MMEs administered for acute surgical pain have a very similar pattern between the elevated circulating catecholamines and those with psychosocial problems, including depression and history of nicotine and alcohol use. Furthermore, we surprisingly found in our findings that those 51 years and younger with elevated circulating levels of catecholamines required significantly higher postoperative and total MMEs during the first 24 hours postoperative period when compared to 51 years and younger with depression and history of nicotine and alcohol use in the control group.

This preliminary study evaluated a potential predictive factor to opioid analgesic responses using translational research methods. These findings have the potential to drive clinical decision-making in acute surgical pain management. Of note, predicting the responses through identification of biologic factors may potentially tailor the decision whether to use opioids as a short-term pain therapy. This is critical for mitigating opioid use disorders in the future.

The findings of this study suggested that future research should focus on the correlation of catecholamines, pain perception, and responses to pain medication. In particular, larger sample size clinical studies that focus on novel strategies to develop precision pain protocols for the management of acute surgical pain. Previous reports have underscored the critical nature of such research [25,26].

There are several limitations to our study. Despite of the fact that we matched for several factors including type of surgery, the impact of surgical trauma, even in the same type of surgery, can significantly impact postoperative outcomes and therefore affect pain management.

Pre-existing conditions were not taken into account if they appeared in the electronic health record. In addition, it must be highlighted that the assessment of severity of pre-existing conditions was not defined by parameters or surrogate outcomes. The quantitative measurement of the severity of these pre-conditions is to be accounted in future research to evaluate other factors in this regards. Other intraoperative and postoperative pain management protocols, with volatile anesthetic agents and non-opioid drugs, may create bias in pain responses in the postoperative pain responses to medications. However, the investigation of this bias was out of the scope of this study. In addition, the sample size utilized for this study was considered very small. This can significantly affect the statistical analysis. It is important to mention that it was our objective to evaluate the responses to opioid analgesics in acute surgical pain when catecholamines were circulating in elevated levels in the population, with a preliminary study design in a clinical scenario. Therefore, this approach was selected and considered feasible at this phase of investigation. Another limitation of this study was that psychosocial conditions are assumed to be associated with elevated catecholamine levels and increased incidence of hypertension in the population. In this study, psychosocial conditions including depression and stress were not assessed in the control group, which can be determinant when measuring opioid drug requirements.

## Conclusion

This preliminary study has used translational research methods to evaluate the potential impact of elevated circulating plasma catecholamines on the response to opioids in acute surgical pain management. We conclude that elevated circulating levels of catecholamines and secondary hypertension predisposed patients to receive higher intraoperative MMEs. In addition, intraoperative midazolam may potentially influence higher total MMEs during the first 24 hours postoperative period.

Based on the results of this study, we conclude that individuals with elevated plasma catecholamine levels had similar trends in consumption of opioid for surgical pain management when compared to those acknowledged to have altered pain perception and pain responses, secondary to pre-existing psychosocial conditions, including depression and history of nicotine and alcohol use.

Though, age was not a predictive factor considered in this study, it is important to emphasize that subjects 51 years and younger, with elevated plasma catecholamine levels, are subject to further research on total opioid consumption for acute surgical pain management.

We strongly believe that this study shows the need for future investigations on catecholamines and opioid consumption, as it relates to putting patients at risk of significant long-term sequela, secondary to the short-term opioid requirements for postoperative pain management.
